# Maternal Thyroid Dysfunction and Risk of Seizure in the Child: A Danish Nationwide Cohort Study

**DOI:** 10.1155/2013/636705

**Published:** 2013-07-28

**Authors:** Stine Linding Andersen, Peter Laurberg, Chun Sen Wu, Jørn Olsen

**Affiliations:** ^1^Department of Endocrinology, Aalborg University Hospital, Søndre Skovvej 15, 9000 Aalborg, Denmark; ^2^Section for Epidemiology, Department of Public Health, Aarhus University, 8000 Aarhus C, Denmark

## Abstract

Thyroid hormones are essential for brain development, and maternal thyroid disease may affect child neurocognitive development. Some types of seizures may also depend upon early exposure of the developing central nervous system, and we hypothesized that maternal thyroid dysfunction could increase the risk of seizure in the child. In a Danish population-based study we included 1,699,693 liveborn singletons, and from the Danish National Hospital Register we obtained information on maternal diagnosis of hyper- or hypothyroidism and neonatal seizure, febrile seizure, and epilepsy in the child. Maternal diagnosis of thyroid dysfunction before or after birth of the child was registered in two percent of the singleton births. In adjusted analyses, maternal hyperthyroidism and hypothyroidism first time diagnosed after birth of the child were associated with a significant increased risk of epilepsy in the child. Moreover, hypothyroidism diagnosed after birth of the child was associated with a significant increased risk of neonatal and febrile seizures. No significant association was seen for maternal diagnosis prior to birth of the child. We speculate if some degree of maternal thyroid dysfunction was already present during the pregnancy in mothers diagnosed after birth of the child and if this untreated condition may present a neurodevelopmental risk.

## 1. Introduction

Thyroid hormones are essential for fetal brain development [1, 2] and required for normal myelination, the regulation of cell migration in the cerebral cortex, hippocampus and cerebellum, the differentiation of neurons, oligodendrocytes, astrocytes and microglia, and synaptogenesis [3]. Thyroid hormone receptors are expressed in the developing human fetal brain during the first trimester of pregnancy [4, 5] before the onset of fetal thyroid hormone production. Thus, maternal thyroid hormones are the key source of thyroid hormone supply to the fetus in the first half of pregnancy, and they also have an important role in fetal brain development in the second half of pregnancy as illustrated by paucity of neurological symptoms in congenital hypothyroidism with failure of the fetal thyroid gland [6]. 

Thyroid diseases are common in women of reproductive age either as overt or subclinical disease [7, 8], and several studies addressed the impact of maternal thyroid dysfunction on child neurocognitive development [9–12]. Seizures are one of the manifestations of neurological disorders and result from an abnormal or excessive discharge of a set of neurons in the brain [13]. Seizures may depend upon early central nervous system (CNS) alterations [14, 15], and the highest incidence occurs during the first year of life [16]. Seizures in the neonatal period are important to recognize as they often relate to an underlying neurotoxic exposure that might adversely affect the developing brain [16]. After the neonatal period, febrile seizures are the most common type of seizures in childhood affecting 2–5% of children [17]. Epilepsy is characterized by recurrent seizures and is the most common serious neurologic disorder in childhood [15]. The aetiology of febrile seizures and epilepsy is only partly understood, but environmental and genetic risk factors have been proposed [15, 17]. 

We hypothesized that maternal thyroid dysfunction, hyper- and hypothyroidism, would increase the risk of seizure in the child via subtle changes in brain structure. We examined the impact of maternal thyroid dysfunction diagnosed before, during, and after the pregnancy on the risk of neonatal seizure, febrile seizure, and epilepsy in the child. More specifically we hypothesized that an association between maternal thyroid dysfunction and the risk of seizure in the child could be caused by one or more of the following mechanisms. (1) *Fetal Programming*. Abnormal thyroid hormone levels or treatment of a thyroid disease before/during pregnancy could affect the fetal brain and increase susceptibility to seizure in the child. An association with maternal thyroid disease diagnosed before birth of the child might then be expected. (2) *Genetics*. A genetic component passed on to the child from the parent suffering from thyroid disease could increase susceptibility to seizure in the child. If genetic factors play a role, an association with paternal thyroid disease or with maternal thyroid disease diagnosed after birth of the child might then also be expected. (3) *Subclinical Thyroid Disease*. The impact of maternal thyroid dysfunction present before and/or during pregnancy but clinically diagnosed and treated after birth of the child could increase susceptibility to seizure in the child. An association with maternal thyroid disease diagnosed after birth of the child might then be expected.

## 2. Materials and Methods

### 2.1. Study Population and Design

We conducted a population-based cohort study using Danish nationwide registers. A unique ten-digit personal identification number is used in all national registers in Denmark, and in the Danish Civil Registration System [18] we identified all liveborn singletons in Denmark between January 1, 1978, and November 30, 2006. 

The Danish National Hospital Register (DNHR) [19] holds data on all admissions to any Danish Hospital since 1977 and all hospital outpatient visits since 1995. For every admission the register contains date of admission and discharge and diagnoses classified according to the 8th revision of the International Classification of Disease (ICD-8) from 1977 to 1993 and the 10th revision (ICD-10) from 1994 and onwards. We included in- and outpatient visits with a main or additional first time diagnosis (see choice of ICD codes below) before January 1, 2007. The “onset” of disease was defined as the day of admission to hospital, and onset of maternal disease was categorized as before/after birth of the child and during pregnancy. The pregnancy period was estimated by subtracting gestational age at birth from the date the child was born.

### 2.2. Diagnosis of Thyroid Dysfunction

Diagnoses of maternal thyroid dysfunction were obtained from DNHR. Hyperthyroidism was defined as ICD-8: 242.00–242.29 and ICD-10: E05–E05.9 (excluding thyrotoxicosis factitia (E05.4), overproduction of thyroid-stimulating hormone (E05.8A), and thyrotoxic heart disease (E05.9A)). Hypothyroidism was defined as ICD-8: 243.99, 244.00–244.09 (excluding secondary hypothyroidism 244.02) and ICD-10: E03–E03.9 and E89.0 (excluding unspecified congenital goitre (E03.0A) and atrophy of the thyroid (congenital E03.1B, acquired E03.4)).

### 2.3. Diagnosis of Seizure

Diagnoses of seizure were obtained from DNHR using the following ICD codes: febrile seizure ICD-8: 780.21 and ICD-10: R56.0 and R56.0A, epilepsy ICD-8: 345.09–345.99 and ICD-10: G40.0–G41.9, and unspecified seizure ICD-8: 780.29 and ICD-10: R56.8. Diagnoses of breath-holding spells (ICD-8 780.20 and ICD-10 R56.8C) were not included. 

Neonatal seizure was defined as ICD-10: P90.9 or any of the seizure diagnoses listed above obtained in the neonatal period (first 28 days of life). Febrile seizure was defined as a diagnosis of febrile seizure after the neonatal period and before the age of 5 years with no previous diagnosis of neonatal seizure or epilepsy. Finally, epilepsy was defined as a diagnosis of epilepsy after the neonatal period [13].

### 2.4. Covariates

 From the Medical Birth Registry [20], which has been computerized since 1973, we obtained information on gender of the child, gestational age at birth, birth weight, 5 minutes Apgar score, and maternal parity and age at birth of child. 

From Statistics Denmark we obtained information on maternal cohabitation, income, origin, and geographical residence at birth of child. Information was only available from 1980, and data from 1980 were used to substitute missing values in 1978 and 1979. For maternal cohabitation and origin we replaced additional missing values by available information in the preceding or following five (origin) or three (cohabitation) years, whichever came first. Maternal residence at birth of child was used as an indicator variable for iodine intake. Iodine is required for thyroid hormone synthesis, and Denmark was previously iodine deficient with regional differences: moderate iodine deficiency in West Denmark and mild iodine deficiency in East Denmark (divided by the Great Belt). The mandatory iodine fortification of salt was introduced in the year 2000 and had increased urinary iodine to lower recommended level in 2004-2005 [21].

From DNHR we obtained information on parental diagnosis of epilepsy and febrile seizure, maternal diagnosis of preeclampsia/eclampsia (ICD-8: 637.03–637.19 and ICD-10: O14-O15.0) and diabetes (ICD-8: 249.00–250.09 and ICD-10: E10.0–E14.9 and O24–O24.9) and child diagnosis of cerebral palsy (ICD-8: 343.99 and 344.99 and ICD-10: G80–80.9) and congenital malformations (ICD-8: 740.99–759.99 and ICD-10: Q00–Q99). 

We excluded children with missing information on maternal identification number *n* = 13 or maternal covariates: parity *n* = 169, origin *n* = 5,015, cohabitation *n* = 1,257, and income *n* = 1,335, thus leaving 1,699,693 singletons in the final cohort for followup. Singletons not alive after the neonatal period (*n* = 838) or who had emigrated (*n* = 109) during the neonatal period were not included in the followup study on the risk of febrile seizure and epilepsy.

## 3. Statistical Analyses

Children were followed up from birth (neonatal seizure) or from day 29 after birth (febrile seizure and epilepsy) and until the date of admission for the first in- or outpatient visit with a diagnosis of seizure, emigration, or death or until day 28 after birth (neonatal seizure), age of 5 years (febrile seizure), or December 31, 2006 (epilepsy).

The Cox proportional hazards model was used to estimate hazard ratio (HR) with 95% confidence interval (95% CI) for risk of neonatal seizure, febrile seizure, and epilepsy in children exposed to maternal hyper- or hypothyroidism diagnosed before and/or after birth of the child compared to children unexposed. The proportional hazards assumption was tested in plots of log cumulative hazards. Robust standard errors were used to adjust for dependence between maternal multiple pregnancies. Univariate and multivariate analyses were performed, and in the multivariate analyses we adjusted for potential confounders including gender of the child (boy/girl), birth year (<1980, 1980–1982, 1983–1985, 1986–1988, 1989–1991, 1992–1994, 1995–1997, 1998–2000, 2001–2003, 2004–2006), and the following maternal variables obtained at the time of the child's birth: age (<20, 20–24, 25–29, 30–34, 35–39, ≥40 years), parity including index pregnancy (1, 2, 3, ≥4), cohabitation (married/not married), income (1st, 2nd, 3rd, 4th quartile), origin (born in Denmark/not born in Denmark), residence (East/West Denmark), and maternal diagnosis of febrile seizure and/or epilepsy registered in the Danish National Hospital Register (DNHR) before January 1, 2007 (yes/no). In parallel analyses, HR with 95% CI for risk of seizure in children exposed to paternal thyroid dysfunction was estimated. 

Apgar score, birth weight, and gestational age were a priori considered possible intermediates and hence not included in the main model. Analyses were repeated after the exclusion of (a) children with a diagnosis of cerebral palsy, congenital malformations, and/or 5-minute Apgar score below 7 (*n* = 173,440), (b) children born to mothers with a diagnosis of preeclampsia/eclampsia and/or diabetes (*n* = 126,812), (c) children with gestational age at birth <37 or >41 weeks or birth weight not appropriate for gestational age (below 10th or above 90th percentile for gestational week and gender) (*n* = 505,397), and (d) children with a diagnosis of thyroid disease (*n* = 2,391). 

Potential confounding by maternal smoking was evaluated in the cohort of children born after December 31, 1995, when information on maternal smoking during pregnancy was available in DNHR. Statistical analyses were performed using STATA version 11 (Stata Corp., College Station, Texas, USA) and a 5% level of significance was chosen. The study was approved by the Danish Data Protection Agency.

## 4. Results

### 4.1. Maternal Diagnosis of Thyroid Dysfunction in relation to Pregnancy

Among 1,699,693 liveborn singletons in Denmark between January 1, 1978, and November 30, 2006, we identified 34,582 singletons (2.0%), whose mother had a diagnosis of hyperthyroidism (1.3%) or hypothyroidism (0.7%) registered in DNHR before January 1, 2007.  Table 1 presents characteristics of children and mothers at birth of the child according to maternal diagnosis of thyroid dysfunction before or after birth of the child.

The majority of exposed singletons were born to mothers diagnosed with hyper- or hypothyroidism after birth of the child (74.3%) with a median time from birth of the child to maternal diagnosis of 10.7 years (range 0.003–28.9 years). For maternal diagnosis before or at birth of the child, median time from diagnosis to birth of the child was 3.0 years (0–27.5 years) for hyperthyroidism and 1.7 years (0–29.8 years) for hypothyroidism. 

**Figure 1:**
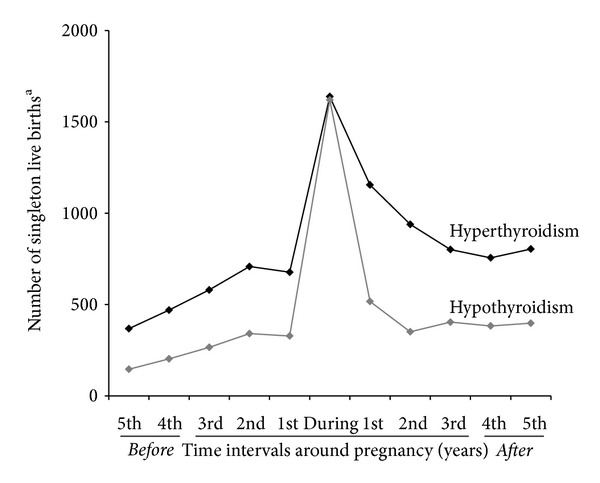
First-time diagnosis of maternal thyroid dysfunction in relation to birth of child. The figure illustrates number of *singleton live births *per year with a first-time diagnosis of maternal hyper- or hypothyroidism registered in the Danish National Hospital Register (DNHR) during the pregnancy (during) and in 1-year intervals before/after the pregnancy (1st, 2nd, 3rd, etc.). ^a^Singleton live births with missing information on gestational age or registration of gestational age <20 or >45 weeks not included (*n* = 60,352). Number of singleton live births with maternal diagnosis during the pregnancy estimated per year.

**Figure 2:**
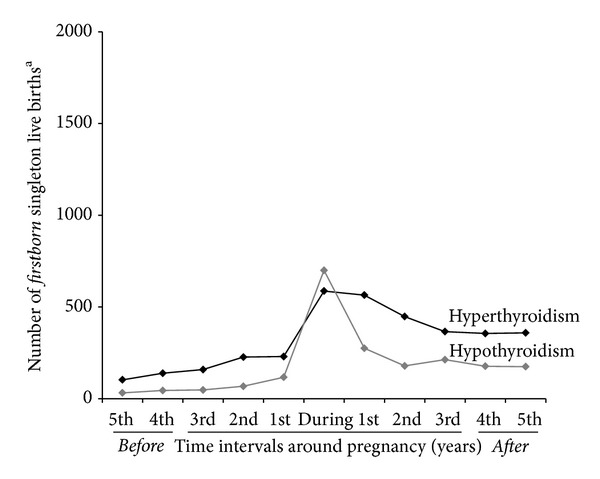
First-time diagnosis of maternal thyroid dysfunction in relation to birth of firstborn child. The figure illustrates number of *firstborn singleton live births* per year with a first-time diagnosis of maternal hyper- or hypothyroidism registered in the Danish National-Hospital Register (DNHR) during the pregnancy (during) and in 1 year intervals before/after the pregnancy (1st, 2nd, 3rd, etc.). ^a^Firstborn refers to primiparous mother. Firstborn singleton live births with missing information on gestational age or registration of gestational age <20 or >45 weeks not included (*n* = 30,363). Number of singleton live births with maternal diagnosis during the pregnancy estimated per year.

In the group of singletons born to mothers with a diagnosis of thyroid dysfunction before birth of the child and valid information on gestational age, 2,498 singleton births followed a maternal first time diagnosis of thyroid disease during the pregnancy. Figures 1 and 2 illustrate the distribution of singleton births with a diagnosis of maternal hyper- or hypothyroidism during pregnancy and in 1 year intervals before and after pregnancy; the relative frequency of maternal diagnosis during pregnancy was higher for hypothyroidism in comparison to hyperthyroidism. 

### 4.2. Main Analyses

**Figure 3:**
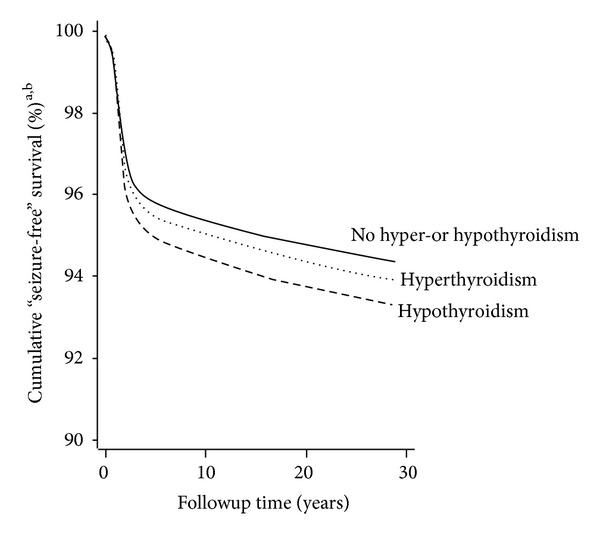
Cumulative percentages of children with no diagnosis of seizure (neonatal seizure, febrile seizure, or epilepsy) by follow-up time from birth of the child and maternal diagnosis of thyroid dysfunction (none, hyperthyroidism, hypothyroidism). ^a^Cox regression model. Outcome: a diagnosis of seizure (neonatal seizure, febrile seizure, or epilepsy). Exposure: maternal diagnosis of hyperthyroidism, hypothyroidism, or no maternal thyroid dysfunction. Diagnoses were registered in the Danish National Hospital Register before January 1, 2007. Model included gender of the child (boy/girl), birth year (<1980, 1980–1982, 1983–1985, 1986–1988, 1989–1991, 1992–1994, 1995–1997, 1998–2000, 2001–2003, 2004–2006), and the following maternal variables obtained at the time of the child's birth: age (<20, 20–24, 25–29, 30–34, 35–39, ≥40 years), parity including index pregnancy (1, 2, 3, ≥4), cohabitation (married/not married), income (1st, 2nd, 3rd, 4th quartile), origin (born in Denmark/not born in Denmark), residence (East/West Denmark), and maternal diagnosis of febrile seizure and/or epilepsy registered in the Danish National Hospital Register (DNHR) before January 1, 2007 (yes/no). ^b^317 cases were censored (febrile seizure *n* = 89, epilepsy *n* = 224, and epilepsy and febrile seizure *n* = 4), all because of emigration. Distribution of the censored cases by exposure group: no hyper- or hypothyroidism (*n* = 306), hyperthyroidism (*n* = 8), and hypothyroidism (*n* = 3).

Children were followed up for up to 28.9 years, and Figure 3 illustrates the cumulative percentages of children with no diagnosis of seizure according to follow-up time and maternal thyroid dysfunction.  Table 2 presents the number of children diagnosed with seizure during followup. Overall, a diagnosis of seizure (neonatal seizure, febrile seizure, and/or epilepsy) occurred more often among children whose mother had a diagnosis of hyper- or hypothyroidism (5.3%) than among unexposed children (4.6%), *P* < 0.001. Median age at diagnosis was 1.4 years for febrile seizure and 5.3 years for epilepsy, and in 18.2% of the cases a diagnosis of epilepsy occurred during the first year of life. 

The crude and adjusted HR for risk of seizure in the child differed according to seizure type and maternal thyroid dysfunction (Table 2). The adjusted model only changed the estimates slightly; whereas maternal hypothyroidism first time diagnosed before or after birth of the child was associated with increased risk of neonatal seizure, febrile seizure, and epilepsy, maternal hyperthyroidism only showed a statistically significant association with epilepsy.

### 4.3. Time of Maternal Diagnosis

**Figure 4:**
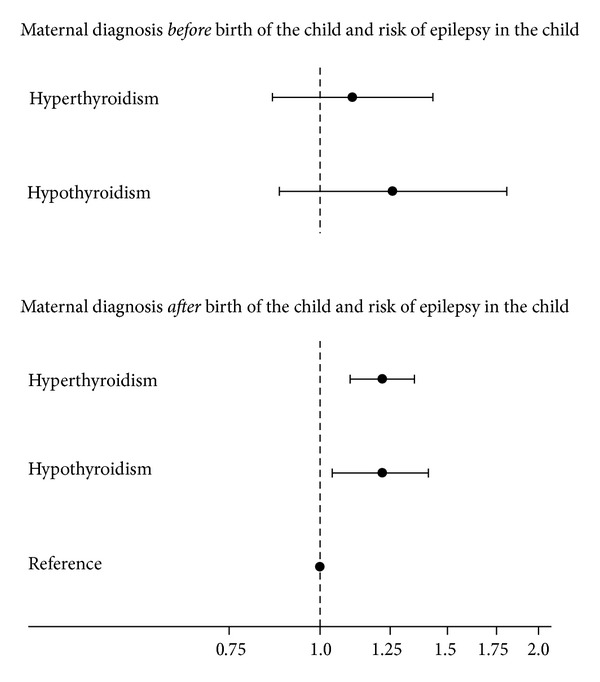
Adjusted hazard ratio with 95% confidence interval for risk of epilepsy in singletons exposed to maternal hyper- or hypothyroidism first time diagnosed before or after birth of the child and registered in the Danish National Hospital Register (DNHR) before January 1, 2007. Reference is children born to mothers with no diagnosis of hyper- or hypothyroidism registered in DNHR before January 1, 2007. Model included gender of the child (boy/girl), birth year (<1980, 1980–1982, 1983–1985, 1986–1988, 1989–1991, 1992–1994, 1995–1997, 1998–2000, 2001–2003, 2004–2006), and the following maternal variables obtained at the time of the child's birth: age (<20, 20–24, 25–29, 30–34, 35–39, ≥40 years), parity including index pregnancy (1, 2, 3, ≥4), cohabitation (married/not married), income (1st, 2nd, 3rd, 4th quartile), origin (born in Denmark/not born in Denmark), residence (East/West Denmark), and maternal diagnosis of febrile seizure and/or epilepsy registered in the Danish National Hospital Register (DNHR) before January 1, 2007 (yes/no).

We subsequently evaluated if the increased risk of epilepsy in children born to mothers with a diagnosis of hyper- or hypothyroidism differed according to time of maternal diagnosis in relation to birth of the child (Figure 4). Maternal hyper- and hypothyroidism first time diagnosed after birth of the child were associated with an increased risk of epilepsy in the child. For maternal diagnosis prior to birth of the child, the estimated HR revealed rather similar increased risk. However, the number of cases was smaller and the confidence intervals were wider. Thus, the associations were not statistically significant.

**Figure 5:**
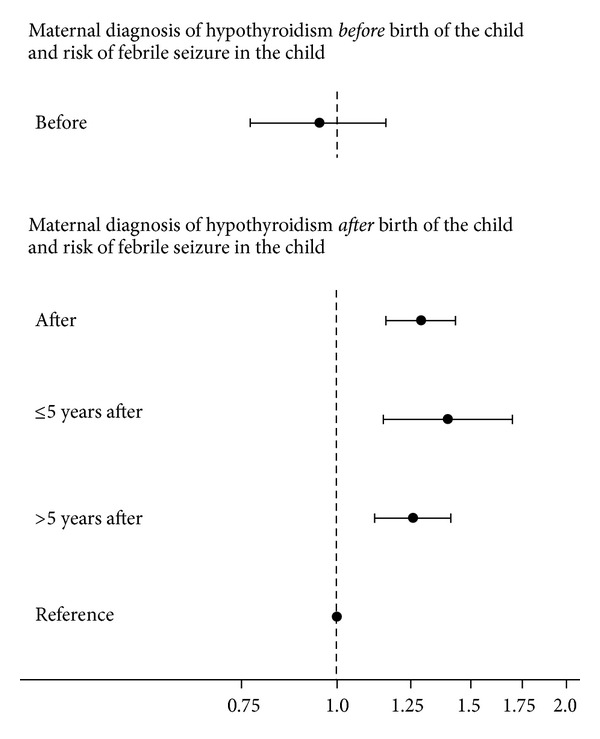
Adjusted hazard ratio with 95% confidence interval for risk of febrile seizure in singletons exposed to maternal hypothyroidism first time diagnosed before or after birth of the child and registered in the Danish National Hospital Register (DNHR) before January 1, 2007. Reference is children born to mothers with no diagnosis of hyper- or hypothyroidism registered in DNHR before January 1, 2007. Model included gender of the child (boy/girl), birth year (<1980, 1980–1982, 1983–1985, 1986–1988, 1989–1991, 1992–1994, 1995–1997, 1998–2000, 2001–2003, 2004–2006), and the following maternal variables obtained at the time of the child's birth: age (<20, 20–24, 25–29, 30–34, 35–39, ≥40 years), parity including index pregnancy (1, 2, 3, ≥4), cohabitation (married/not married), income (1st, 2nd, 3rd, 4th quartile), origin (born in Denmark/not born in Denmark), residence (East/West Denmark), and maternal diagnosis of febrile seizure and/or epilepsy registered in the Danish National Hospital Register (DNHR) before January 1, 2007 (yes/no).

Maternal hypothyroidism was associated with an increased risk of febrile seizure and neonatal seizure in the child.  Figure 5 illustrates the stratified analyses for febrile seizure in the child according to time of maternal diagnosis of hypothyroidism. Only maternal diagnosis after birth of the child was associated with significantly increased risk of febrile seizure in the child, and this applied to maternal diagnosis of disease both within 5 years and more than 5 years after birth of the child. For neonatal seizure in the child, both maternal diagnosis prior to and after birth of the child revealed an increased risk, but results only reached statistical significance in children born to mothers diagnosed after birth of the child (diagnosis before birth of the child: 1.65 (0.88–3.06), after birth of the child: 1.83 (1.27–2.65)).

Among the 2,498 children whose mother had hyper- or hypothyroidism diagnosed during the pregnancy, a total of 111 children developed seizure during followup (neonatal *n* = 8, febrile *n* = 83, and epilepsy *n* = 26), and the adjusted HR for risk of seizure in the child did not indicate that maternal diagnosis during pregnancy revealed a high risk: neonatal seizure HR 1.64 (95% CI 0.82–3.28), febrile seizure 1.10 (0.88–1.36), and epilepsy 1.25 (0.85–1.84). However, numbers were small and did not permit stratification by maternal type of thyroid dysfunction.

### 4.4. Paternal Thyroid Dysfunction

We identified 5,742 singletons (0.3%) whose father had a diagnosis of hyperthyroidism (*n* = 3,981) or hypothyroidism (*n* = 1,761) registered in DNHR before January 1, 2007, and among these children, 288 (5%) had a diagnosis of seizure (neonatal *n* = 9, febrile *n* = 190, and epilepsy *n* = 109). No significant increased risk of seizure in children exposed to paternal thyroid disease was observed (adjusted HR neonatal seizure 0.87 (95% CI 0.45–1.68), febrile seizure 0.99 (0.86–1.15)). The adjusted HR for risk of epilepsy in the child was on the borderline of statistical significance 1.20 (95% CI 0.99–1.46). 

### 4.5. Sensitivity Analyses

The exclusion of children with a diagnosis of cerebral palsy, congenital malformations, and/or Apgar score below 7 revealed almost identical adjusted HR to the nonrestricted analyses. However, after such exclusion, the association between maternal hypothyroidism and the risk of neonatal seizure in the child was no longer statistically significant (adjusted HR 1.34 (0.83–2.16)). Restricting the cohort to children born at term with a birth weight appropriate for gestational age did not change results for maternal hyperthyroidism, but for hypothyroidism the risk of neonatal seizure (adjusted HR 1.42 (0.87–2.32)) and epilepsy (1.18 (0.98–1.43)) were no longer statistically significant.

The exclusion of children born to mothers with a diagnosis of preeclampsia and/or diabetes did not change results. A small group of children had a diagnosis of thyroid disease (hyperthyroidism *n* = 960, hypothyroidism *n* = 1,431) with a median age at diagnosis of 14.6 years. The frequency of seizure was higher in this group (8.4%), but the associations were similar after the exclusion of these children. Restricting analyses to firstborn child did not change results, neither did the exclusion of children born to foreign-born mothers. We identified 627,100 singletons with available information on maternal smoking during the pregnancy. Applying the same main model to this restricted cohort revealed similar associations regardless of the inclusion of maternal smoking in the model (data not shown).

## 5. Discussion

### 5.1. Principle Findings

Based on Danish nationwide registers we found an increased risk of seizure in children born to mothers diagnosed with thyroid dysfunction in a Danish hospital after birth of the child. Maternal hypothyroidism increased the risk of neonatal seizure, febrile seizure, and epilepsy, whereas maternal hyperthyroidism solely revealed an increased risk of epilepsy in the child. No significant association with maternal thyroid dysfunction diagnosed prior to birth of the child or with paternal thyroid dysfunction was observed.

### 5.2. Brain Development, Thyroid Hormones, and Risk of Epilepsy

Thyroid hormones are essential for fetal brain development [2] and involved in a number of developmental events including regulation of cell migration in the cerebral cortex, hippocampus, and cerebellum and the differentiation of neurons, oligodendrocytes, astrocytes, and microglia [3]. Disruptions in the normal process of cerebral cortex development and in glial function have been proposed as some of the underlying causes of epilepsy [14]. Malformations of cortical development are macro- or microscopic abnormalities of the cerebral cortex that may arise during cortical development in the first or second trimester of pregnancy and may predispose to epileptic seizures [22]. Glia cells are nonneuronal cells involved in neuronal functions and the maintenance of tissue homeostasis, and it has been proposed that especially astrocytes and microglia dysfunction may contribute to the pathophysiology of epilepsy [23].

It is intriguing how both lack and excess of maternal thyroid hormones can increase the risk of epilepsy in the child, but experimental studies in rats provide evidence that both maternal hyper- and hypothyroidism may interfere profoundly with early fetal brain development [24].

### 5.3. Risk of Neonatal and Febrile Seizures

Maternal hypothyroidism in contrast to hyperthyroidism was associated with increased risk of neonatal and febrile seizures in the child. Febrile seizures are the most common seizures in childhood and most often benign [25]. The aetiology is only partly understood although genetic and environmental risk factors have been described [25]. Concerning febrile seizures and brain development, controversies exist on the possible association with hippocampal sclerosis and atrophy [17]. It has been brought forward that structural changes in the hippocampus might either preexist and predispose to febrile seizures or result from prolonged febrile seizures and increase the long-term risk of epilepsy [17, 26]. Concerning a possible association with maternal hypothyroidism, evidence suggests that the hypothyroid state during brain development may induce structural and functional alterations in the hippocampus [27], and one study reported that children with congenital hypothyroidism on regular Levothyroxine substitution were less prone to develop febrile seizures [28]. 

Neonatal seizures often relate to an underlying cause, which is most often perinatal hypoxia-ischemia, but also stroke, haemorrhage, infections, or metabolic disorders [16]. The association between maternal hypothyroidism and risk of neonatal seizure was no longer significant when excluding children with a diagnosis of cerebral palsy, congenital malformations, or low Apgar score or when restricting the analyses to children born at term with a birth weight appropriate for gestational age. These factors might constitute intermediates on the causal pathway [29–31], and hence adjustment could induce bias including collider bias [32]. Alternatively, the lack of a significant association after such exclusion could be due to the reduced size of the study population.

### 5.4. Time of Maternal Diagnosis in relation to Birth of the Child

The increased risk of seizure in children born to mothers with a later diagnosis of thyroid dysfunction could relate to disturbances in fetal brain development secondary to abnormal maternal thyroid hormone levels. We only found a significant association in children born to mothers diagnosed after birth of the child. Low-grade thyroid dysfunction may be present for years before diagnosis [33], and these children could have been exposed to maternal subclinical thyroid disease present during the pregnancy. On the other hand, children born to mothers diagnosed before or during the pregnancy might not have been exposed to abnormal maternal thyroid hormone levels to the same extent since proper treatment of maternal disease is expected to have been initiated. Another possible explanation of the association with maternal thyroid dysfunction diagnosed after birth of child could be a genetic component linking maternal thyroid dysfunction and risk of seizure in the child; however, no significant association with paternal thyroid disease was observed. Finally, we cannot exclude if the lack of an association with maternal thyroid dysfunction diagnosed before birth of child and with paternal thyroid dysfunction was due to the relatively small number of children in these groups and consequently lack of power to detect an existing difference with statistical significance.

### 5.5. Strengths and Limitations

The strength of our study is the large study population and long follow-up period with virtually complete followup. Thus, bias due to selection of study participants is unlikely. We are aware of the risk of violating the principles of “not conditioning on the future” (exposure registered after outcome has occurred) when analysing the association with maternal thyroid disease diagnosed after birth of the child. The time from birth of child to maternal diagnosis becomes “immortal”, which may cause bias [34]. However, we estimate this bias to be small due to the low mortality during followup. 

The quality of the diagnoses in DNHR has been evaluated, and the positive predictive value of a diagnosis of febrile seizure and epilepsy was 93 and 81%, respectively [35, 36]. A diagnosis of thyroid disease, not particularly in pregnancy but in the general population, only revealed misclassification in 2% of the cases [37]. We were able to distinguish between maternal hyper- and hypothyroidism, but further classification of maternal disease and/or epilepsy was not possible as the validity decreases when the number of digits in the ICD-code increases [38].

Misclassification of exposure and outcome does exist in our study population; however, we believe this misclassification is nondifferential. Maternal onset of disease was defined as the day of first admission to hospital. We only included women with a hospital diagnosis of thyroid disease, and we did not have results of thyroid function tests, thyroid autoantibodies, scintigraphy, and/or treatment modality. We believe that pregnant women are in general referred to hospital for diagnosis and therapy [39], but they might present with symptoms and even have therapy initiated in general practice years before referral to hospital. On the other hand, we cannot exclude that management of maternal thyroid disease first time diagnosed after birth of child could take place in general practice alone. In our study population, maternal hyperthyroidism was more frequent than hypothyroidism. This might be due to different referral pattern for hyper- and hypothyroidism. In addition, the Danish population was iodine deficient during part of the study period, and in this period there were relatively more hyperthyroidism and less hypothyroidism in the population [40]. A first-time diagnosis of maternal thyroid disease during pregnancy occurred relatively more often in hypothyroidism compared to hyperthyroidism. This finding seems likely, as hyperthyroidism in women of reproductive age might present with obvious symptoms leading to diagnosis, whereas hypothyroidism might not be detected until symptoms exaggerate during pregnancy or if thyroid function parameters are screened. 

Although we adjusted for a number of potential confounders, unmeasured confounding might still exist. Information on maternal infections and use of antibiotic during pregnancy [41] was not included as these variables are not expected to be related to our exposure.

### 5.6. Perspective

The potential benefits and harms of screening pregnant women for thyroid dysfunction in early pregnancy have been an ongoing discussion in recent years, as discussed in detail in [42]. The present study adds further evidence to this discussion, and if future studies corroborate that many pregnant women suffer from undiagnosed thyroid dysfunction and that this increases the risk of neurocognitive diseases in the child, screening may be indicated as suggested by several authors [43, 44].

## 6. Conclusions

Maternal hyper- and hypothyroidism diagnosed after birth of the child may increase the risk of epilepsy in the child. In addition, maternal hypothyroidism diagnosed after birth of child was associated with increased risk of neonatal seizure and febrile seizure. The findings lead to speculations whether undetected maternal thyroid hormone aberrations were already present during the pregnancy in mothers diagnosed after birth of the child.

## Figures and Tables

**Table 1:** Characteristics of singletons born in Denmark between January 1, 1978, and November 30, 2006, and their mothers at birth of the child.

	No maternal thyroid dysfunction^a^		Maternal hyperthyroidism^b^				Maternal hypothyroidism^b^			
			Before birth		After birth		Before birth		After birth	
	*n*	%	*n*	%	*n*	%	*n*	%	*n*	%
Singletons	1,665,111	98.0	5,738	0.3	17,139	1.0	3,155	0.2	8,550	0.5
Maternal characteristics										
Parity^c^										
Primipara	759,826	45.6	1,827	31.8	7,804	45.5	1,068	33.8	4,018	47.0
Multipara	905,285	54.4	3,911	68.2	9,335	54.5	2,087	66.2	4,532	53.0
Age (years)										
<25	378,535	22.7	518	9.0	4,523	26.4	309	9.8	2,076	24.3
25–35	1,155,045	69.4	4,241	73.9	11,277	65.8	2,325	73.7	5,757	67.3
>35	131,531	7.9	979	17.1	1,339	7.8	521	16.5	717	8.4
Origin										
Born in Denmark	1,520,213	91.3	5,090	88.7	15,556	90.8	2,698	85.5	7,497	87.7
Not born in Denmark	144,898	8.7	648	11.3	1,583	9.2	457	14.5	1,053	12.3
Residence^d^										
West Denmark	939,188	56.4	3,329	58.0	10,621	62.0	1,610	51.0	4,688	54.8
East Denmark	725,923	43.6	2,409	42.0	6,518	38.0	1,545	49.0	3,862	45.2
Child characteristics										
Gender										
Boy	854,831	51.3	2,884	50.3	8,665	50.6	1,615	51.2	4,429	51.8
Girl	810,280	48.7	2,854	49.7	8,474	49.4	1,540	48.8	4,121	48.2
Gestational age at birth (weeks)^e^										
<37	70,984	4.4	363	6.5	818	5.1	193	6.2	425	5.3
37–41	1,392,330	87.0	4,870	86.7	13,837	86.6	2,713	87.5	6,896	85.5
>41	138,383	8.6	382	6.8	1,328	8.3	196	6.3	740	9.2
Birth weight (grams)^e^										
Mean (SD)	3488 (559)		3442 (591)		3425 (567)		3546 (596)		3492 (584)	
5 minutes Apgar score^f^										
7–10	1,635,867	99.2	5,625	99.0	16,854	99.3	3,096	99.1	8,415	99.4
0–6	12,554	0.8	55	1.0	118	0.7	27	0.9	49	0.6

^a^No maternal diagnosis of hyper- or hypothyroidism registered in the Danish National Hospital Register (DNHR) before January 1, 2007.

^b^Maternal first-time diagnosis of thyroid dysfunction (hyper- or hypothyroidism) before or after birth of the child and registered in DNHR before January 1, 2007.

^c^Number of births (live and stillbirths) including index pregnancy.

^d^Divided into regions by the Great Belt: West Denmark with previously moderate iodine deficiency and East Denmark with previously mild iodine deficiency.

^e^Singletons with missing value on gestational age or birth weight, registration of gestational age <20 or >45 weeks or birth weight <500 or >6000 grams not included (*n* = 65,235).

^f^Singletons with missing value on 5-minute Apgar score not included (*n* = 17,033).

**Table 2:** Hazard ratio (HR) with 95% confidence interval (95% CI) for seizure in singletons born in Denmark, 1978–2006, according to maternal diagnosis of hyper- or hypothyroidism registered in the Danish National Hospital Register (DNHR) before January 1, 2007. Reference is singletons born to mothers with no diagnosis of thyroid dysfunction registered in DNHR before January 1, 2007.

	Singletons (*n*)	Crude HR (95% CI)	Adjusted HR (95% CI)^a^	*P* ^ b^
Seizure^c^				
No hyper- or hypothyroidism	77,444	1.00 (reference)	1.00 (reference)	
Hyper- or hypothyroidism	1,844	1.11 (1.06–1.16)	1.12 (1.07–1.18)	<0.001
Hyperthyroidism	1,185	1.06 (1.00–1.12)	1.08 (1.02–1.15)	0.009
Hypothyroidism	659	1.20 (1.11–1.30)	1.20 (1.11–1.30)	<0.001
Neonatal seizure^d^				
No hyper- or hypothyroidism	3,109	1.00 (reference)	1.00 (reference)	
Hyper- or hypothyroidism	89	1.39 (1.13–1.71)	1.42 (1.15–1.75)	0.001
Hyperthyroidism	50	1.18 (0.89–1.56)	1.22 (0.92–1.62)	0.161
Hypothyroidism	39	1.80 (1.31–2.47)	1.78 (1.30–2.44)	<0.001
Febrile seizure^e^				
No hyper- or hypothyroidism	55,798	1.00 (reference)	1.00 (reference)	
Hyper- or hypothyroidism	1,249	1.08 (1.02–1.14)	1.09 (1.03–1.15)	0.003
Hyperthyroidism	779	1.02 (0.95–1.09)	1.03 (0.95–1.11)	0.464
Hypothyroidism	470	1.20 (1.10–1.32)	1.21 (1.10–1.32)	<0.001
Epilepsy^f^				
No hyper- or hypothyroidism	22,173	1.00 (reference)	1.00 (reference)	
Hyper- or hypothyroidism	628	1.25 (1.15–1.35)	1.21 (1.11–1.31)	<0.001
Hyperthyroidism	426	1.24 (1.13–1.37)	1.20 (1.09–1.32)	<0.001
Hypothyroidism	202	1.26 (1.09–1.44)	1.22 (1.06–1.40)	0.005

^a^Adjusted model included gender of the child (boy/girl), birth year (<1980, 1980–1982, 1983–1985, 1986–1988, 1989–1991, 1992–1994, 1995–1997, 1998–2000, 2001–2003, 2004–2006), and the following maternal variables obtained at the time of the child's birth: age (<20, 20–24, 25–29, 30–34, 35–39, ≥40 years), parity including index pregnancy (1, 2, 3, ≥4), cohabitation (married/not married), income (1st, 2nd, 3rd, 4th quartile), origin (born in Denmark/not born in Denmark), residence (East/West Denmark), and maternal diagnosis of febrile seizure and/or epilepsy registered in the Danish National Hospital Register (DNHR) before January 1, 2007 (yes/no).

^b^ *P* value for results of the adjusted model.

^c^Diagnosis of neonatal seizure, febrile seizure, and/or epilepsy.

^d^Diagnosis of seizure in the neonatal period (first 28 days of life).

^e^Diagnosis of febrile seizure after the neonatal period and before the age of 5 years with no previous diagnosis of epilepsy or neonatal seizure.

^f^Diagnosis of epilepsy after the neonatal period and before January 1, 2007. Epilepsy diagnosis following neonatal seizure (*n* = 654), following febrile seizure (*n* = 3,104).
